# 

**Published:** 1877-04-20

**Authors:** 


ORIGINAL AND SELECTED ARTICLES—

A Sketch of the History, Nature, Advantages and Processes of the Modern Improved Turkish Bath ..... 85
How to Manage a case of Labor. By John W. Martin, M. D ..... 86
Veratrum Viride. By C. J. Rademaker ..... 90
Obstetric Memoranda ..... 92
Experimentation on Death from Chloroform ..... 93
Notes in Practice. By C. L. Gregory, M. D ..... 94
The Value of Sneezing in the Reduction of Hernia. By Charles Denison, M D ..... 95
The Management of cases of Cholera Morbus. By Charles C. Pike, M. D ..... 97
Iodine and its Preparations in the Therapeutics of Infancy ..... 98

ABSTRACTS AND GLEANINGS—
Veratrum Viride in Puerperal Convulsions, 98. Solution of Silver in the Treatment of Orchitis, 99 The Distinctions Between the Action of Chloral and Opium, 99. A New Bloodless Operation, 99. New Observations Concerning the Therapeutic Action of Carbolized Camphor, 100. Pills of Sulphate Quinia. 100. Baking Powders, 100. International Medical Congress—Section of Medicine, 101. Treatment of Coxalgia, 101. Anæsthesia, 102. Antidote for Carbolic Acid, 102 Agents Affecting the Secretion of the Bile, 103. The Blue Light Cure, 103. How to Make Leeches Bite, 103. Sulpho Cyanide of Potassium, 103. Thermometry in Diseases, 104. Eau de Cologne as an Anæsthetic, 104 The Indianapolis Sun, 104. Typographical Errors, 104. Tiemann & Co.'s New Thermometer, 104. Aconite in Headache, 105. Chloral Hydrate in Tetanus, 105.

PRESCRIPTIONS AND FORMULÆ—Vomiting in Pregnancy, 105. Chill Tonic, 105. Atonic Dyspepsia and Cardialgia, 105. Amenorrhea in Anæmic Subjects, 105. Hooping Cough, 105. Common Cough Mixture, 106. For Scrofula, 106. Catarrh of the Bladder, 106. Chronic Rheumatism, 106

PRACTICAL NOTES—
Ginger Beer, 106, Swollen Joints from Rheumatism, 106. An Excellent Sedative, 106. For Burns, 105. Falling Hair, 106. Stove Blacking, 106. Toothache 107. Dentrific, 107. Lime Water, 107. Hydro Bromic Ether, 107. For Itching Chilblains, 107. Muscular Rheumatism, 107. Cologne Water, 107. Harness Blacking, 107 For Cracked Skin or Hands, 107. Gasoline, 107.

SCIENTIFIC ITEMS—
The Telephone, 107. A Changeable Star, 107. Plural Births, 107.

EDITORIAL AND MISCELLANEOUS—
To Our Subscribers, 108 Professional Brotherhood, 108. Medical Examining Board, 109. Country Physicians, 109. Arkansas State Medical Association, 109 American Medical Association, 110. The Epizooty, 110. Bellevue College Medical Hospital, 110. Cinchonidia, 110. Turkish Baths, 110.

PAMPHLETS RECEIVED—
The Importance of the Uterine Ebb as a Factor in Pelvic Surgery, 110. The Relations of Medicine to Modern Unbelief, 110. The Quarterly Journal of Inebriety, 110.

